# Cardiac Resynchronization Therapy Reduces Metaboreflex Contribution to the Ventilatory Response in Heart Failure Population

**DOI:** 10.1155/2012/914071

**Published:** 2012-03-20

**Authors:** Jérémie Jaussaud, Laurie Aimable, Pierre Bordachar, Pierre Dos Santos, Laurent Barandon, Philippe Ritter, Raymond Roudaut, Hervé Douard

**Affiliations:** Division of Clinical Cardiology, Hôpital Cardiologique, Hôpital du Haut Lévêque, CHU Hôpitaux de Bordeaux, avenue Magellan, 33600 Pessac, France

## Abstract

*Background*. Metaboreflex overactivation has been proprosed to explain exaggerated hyperventilation in heart failure population. We investigated the metaboreflex activation after cardiac resynchronization therapy (CRT). *Methods*. 10 heart failure patients (mean left ventricular ejection fraction (LVEF) 27 ± 4%) schedulded for CRT implantation were prospectively studied. At baseline and after 6 month follow up two maximal cardiopulmonary exercise tests with and without regional circulatory occlusion (RCO) during recovery were performed. RCO was achieved by inflation of bilateral upper thigh tourniquets 30 mmHg above peak systolic blood pressure during 3 minutes after peak exercise. Metaboreflex contribution to the ventilatory response was assessed as the difference in ventilatory data at the third minute during recovery between the two tests (Δ). *Results*. Patients had enhanced VE/VCO_2_ slope (40 ± 9) and an evident metaboreflex contribution to the high ventilatory response (ΔVE: 3 ± 4 L/min; *P* = 0.05, ΔRR: 4.5 ± 4/min; *P* = 0.003 and ΔVE/VCO_2_: 5.5 ± 4; *P* = 0.007). 6 months after CRT implantation, NYHA class, LVEF, peak VO_2_ and VE/VCO_2_ were significantly improved (1.4 ± 0.5; *P* < 0.001, 42 ± 7%; *P* < 0.001, 16.5 ± 3 mL/kg/min; *P* = 0.003; 33 ± 10; *P* = 0.01). Metaboreflex contribution to VE, RR, and VE/VCO_2_ was reduced compared with baseline (*P* = 0.08, *P* = 0.01 and *P* = 0.4 resp.). *Conclusion*. 6 months after CRT metaboreflex contribution to the ventilatory response is reduced.

## 1. Introduction

Patients suffering from chronic heart failure (HF) have constant impaired exercise response with a reduced peak oxygen consumption and an exaggerated ventilatory response expressed by an increase in the slope relating the minute ventilation (VE) to the carbon dioxide production (VCO_2_) [1]. Nevertheless, pathophysiological basis of such as hyperventilation remain unclear. An important cause of hyperpnea during effort would be the enlargement of physiological dead space and ventilation-perfusion mismatch by alveolar hypoperfusion from hemodynamic dysfunction. Another determinant is the early cardiorespiratory reflex dysregulation. This was evidenced by increased peripheral and central chemosensitivity, impaired sympathovagal balance with sympathetic predominance and depressed baroreflex circulation control [2]. In addition, muscle metaboreflex contribution to the ventilatory response has been extensively investigated in heart failure. Of particular interest, Piepoli et al. found a significant overactivation of the peripheral metaboreflex (group III and IV intramuscular afferents sensitive to metabolic products of skeletal muscle work) leading to an early hyperventilation during exercise and a high VE/VCO_2_ slope in heart failure population [3]. Piepoli's “muscle hypothesis” is explained by structural, metabolic, and perfusion muscular changes. In particular, both a reduction in capillar density and a shift from slow-twitch type 1 muscular fibres to fast-twitch type 2 fibres were previously reported. It leads to depressed oxidative capacity and maximal oxygen consumption by a significant reduction of mitochondrial density and an increase of the glycogenolytic metabolism with a high production of carbon dioxide leading to early anaerobic threshold and high respiratory exchange ratio (RER) at peak of the exercise [4, 5]. These muscular changes and inadequate perfusion of exercising muscles may cause local overproduction and accumulation of muscle metabolic byproducts which trigger stimulation of group III and IV neural afferents (metaboreceptors) [6, 7].

In previous investigation authors found a significant improvement in exercise capacity after cardiac resynchronization therapy (CRT). Of particular interest, we demonstrated significant improvement of maximal oxygen consumption, reduction of the peak RER, and a later anaerobic threshold assessing a significant improvement in oxidative capacity. In addition, ventilatory response by VE/VCO_2_ slope is reduced after CRT. But it remains unclear how CRT could improve the ventilatory response [8–10].

We aim to investigate exercise oxidative capacity, ventilatory response, and metaboreflex activation before and 6 months after CRT implantation. We suggest that ventilatory response improvement is associated with a significant reduction of the metaboreflex activation.

## 2. Methods

Refractory HF patients matching the following criteria were prospectively included in the study: indication for CRT implantation according to current indications (QRS duration >120 ms, LV ejection fraction <35%, NYHA symptom class II/III or IV, and optimal heart failure medical regimen). From 2 days before CRT implantation, patients underwent clinical and echocardiographic evaluation. In addition, they performed two maximal cardiopulmonary exercise testing for an evaluation of the metaboreceptor contribution to ventilatory response in the lower limb by a regional circulatory occlusion (RCO). After 6-month followup, patients underwent similar clinical, echocardiographic and exercise evaluation.

### 2.1. Clinical Evaluation

All patients scheduled for CRT and included in this study underwent evaluation before and 6 months after CRT implantation including a clinical (NYHA class, 6-minute hall-walk test, and a quality of life assessment using Minnesota Living with Heart Failure test) and a 12-lead electrocardiogram for heart rate and QRS duration measurement.

### 2.2. Echocardiographic Measurement

2D echocardiograms were performed using a 5.0 MHz imaging probe connecting to an ultrasound system (Vivid 7, Vingmed-General Electric, Horten, Norway). Echocardiograms were loaded into a computer system (Echopac, GE), and all measurements were obtained for all patients at baseline and 6 months after implantation. Echocardiograms were analyzed by a single experienced sonographer. Sample loops were analyzed offline on an Echopac computer workstation to obtain telediastolic (TDV) and tele-systolic LV (TSV) volumes using the methods of disks. Ejection fraction was calculated as follows: (TDV − TSV)/TDV × 100%.

### 2.3. Testing Procedure and Data Collection

Symptom-limited exercises test with ventilatory expired gas analysis were performed using a cycle ergometer with a 10 watts/minute protocol (Ergocard, Medisoft, Sorinnes, Belgium). Continuous standard 12-lead electrocardiograms, right arm manual blood pressure measurements, and heart rate recordings were monitored at every stage. Data for oxygen consumption (VO_2_), carbon dioxide production (VCO_2_), minute ventilation (VE), respiratory rate (RR), and workload were collected continuously throughout the exercise. Oxygen and carbon dioxide sensors were calibrated using gases with known oxygen, nitrogen, and carbon dioxide concentrations prior to each test.

Ventilatory efficiency during exercise was obtained by the linear regression slope relating VE to VCO_2_ from the beginning to the peak of the effort [11]. The peak circulatory power was measured as the product of the peak VO_2_ and the systolic blood pressure as described previously [12]. The respiratory exchange ratio (RER) at peak was measured using the rapport between the VCO_2_ and the VO_2_. The anaerobic threshold was assessed by the Wasserman's method. During passive recovery VE, RR, and VE/VCO_2_ were collected at 1, 2, and 3 minutes.

### 2.4. Metaboreceptor Test

The evaluation of the metaboreceptor contribution to ventilatory data (VE, RR and VE/VCO_2_) in the lower limb included two exercises (24 hours interval): (a) a maximal exercise test with gas exchange measurement according to the precedent protocol followed by a passive recovery and (b) a maximal exercise test at the same level (duration and work load) followed by a venous and arterial regional circulatory occlusion (RCO) by inflation of bilateral upper thigh tourniquets to 30 mmHg above peak exercise arm systolic pressure. During both recoveries VE, RR, and VE/VCO_2_ were collected at 1, 2 and 3 minutes. After 3 minutes RCO was released. The contribution of the muscle metaboreceptors was evaluated by trapping the metabolites in the exercising muscle after exercise. This protocol has been shown to fix the metabolic state of the muscle and to maintain the activation of the metaboreflex [13, 14]. The metaboreflex contribution to the ventilatory response was defined as the difference (Δ) in VE, RR, and VE/VCO_2_ at the third minute recovery with and without RCO.

### 2.5. CRT Implantation

All patients received a biventricular pacing device for CRT with a right ventricular apical lead and left ventricular (LV) lead positioned through the coronary sinus in an LV epicardial vein. Biventricular pacing programmation including atrioventricular delay was achieved by an experienced physician for optimal hemodynamic results.

### 2.6. 6-Month Followup

A similar evaluation including clinical, echocardiographic, and metaboreflex assessments was performed.

### 2.7. Statistical Analysis

Variables are summarized by mean ± SD. Paired *t*-tests were used to compare differences between parameters at baseline and after CRT within our population. All statistical tests with a *P* value of <0.05 were considered to be significant.

## 3. Results

### 3.1. Clinical and Echocardiographic Parameters at Baseline

Ten patients (age:  62 ± 9  years) were included in this study. Baseline characteristics are summarized in Table 1. Patients had severe depressed left ventricular ejection fraction (LVEF: 27 ± 4%) and significant LV dilation (LV tele-systolic volume: 142 ± 58 mL). Mean NYHA status and six-minute walking test were increased at 2.4 ± 0.5 and  460 ± 84  meters respectively with a mean quality of life score at 24 ± 14. Mean brain natriuretic peptide (BNP) was at 371 ± 182 pg/mL. Mean heart rate and QRS duration were at 69 ± 11/minute and 147 ± 27 ms.

### 3.2. Exercise and Metaboreflex Test at Baseline

 Patients had a reduced peak of VO_2_ at 14 ± 4 mL/kg/min with a high ventilatory response (mean VE/VCO_2_ slope at 39 ± 10). Mean peak workload and exercise duration were at  80 ± 25  watts and  474 ± 156  seconds. The mean peak circulatory power was at 2320 ± 821 mL/kg/min·mmHg. The mean peak RER and the mean time to anaerobic threshold were at 1.31 ± 0.18 and  163 ± 94  seconds. All subjects completed the metaboreceptor protocol without complication. The ventilatory variables increased similarly during the exercises without and with postexercise RCO (*P* > 0.05; Table 2). During recovery, ventilation expressed as VE, RR, and VE/VCO_2_ was higher at the third minute with RCO than without RCO (ΔVE:  3 ± 4  L/min; *P* = 0.05, ΔRR: 4.5 ± 3/min; *P* = 0.003, ΔVE/VCO_2_: 5.5 ± 4; *P* = 0.007) (Table 3). These results assessed the significant metaboreflex contribution to the ventilatory response before CRT.

### 3.3. Clinical and Echocardiographic Parameters after 6-Month Followup

 All patients were implanted with a CRT device without complication and all subjects had a permanent (>99% of the time) biventricular pacing during rest and at peak of exercise. Patients experienced a significant reduction of dyspnea (mean NYHA status 1.4 ± 0.5, *P* = 0.0002) without significant changes of the 6-minute walking test and the quality of life score (527 ± 214 meters, *P* = 0.28 and 21 ± 15, *P* = 0.24, resp.). Mean QRS duration and BNP were reduced to 129 ± 31 ms (*P* = 0.1) and 150 ± 145 pg/mL (*P* = 0.06). In our population we found a nonsignificant increase of the mean consumption of betablocker and ACE inhibitors (*P* = 0.11). In addition, mean LVEF and LV tele-systolic volume were significantly improved to 39 ± 9% (*P* = 0.006) and 87 ± 46 mL (*P* = 0.04).

### 3.4. Exercise and Metaboreflex Test after 6-Month Followup (Tables 3 and 4)

 Mean peak of VO_2_ was significantly improved at 16.5 ± 3 mL/kg/min (*P* = 0.003) with a nonsignificant postponed anaerobic threshold at  195 ± 69  seconds (*P* = 0.06). Mean peak RER was reduced to 1.2 ± 0.1 (*P* = 0.1). The ventilatory response was significantly improved (VE/VCO_2_ slope: 33 ± 10, *P* = 0.01) after CRT. Nevertheless, mean exercise duration and maximal workload were not significantly increased (552 ± 142 seconds; *P* = 0.15 and  93 ± 23  watts). However, hemodynamic condition at the peak of exercise was improved (peak systolic blood pressure: 168 ± 31 mmHg; *P* = 0.01 and peak circulatory power: 2770 ± 791 mL/kg/min·mmHg; *P* = 0.004). After 6-month followup, the VE and the RR were lower at the third minute of recovery with RCO than without RCO. But the VE/VCO_2_ was not significantly increased anymore (ΔVE: −1 ± 5 L/min; *P* = 0.6, ΔRR: −1 ± 3/min; *P* = 0.53, ΔVE/VCO_2_: 3.5 ± 4; *P* = 0.06). The negative effect of circulatory occlusion after 6-month followup suggests the nonsignificant metaboreflex contribution to the ventilatory response after CRT. In addition, the metaboreflex contribution to RR was significantly reduced (*P* = 0.01) after CRT. Nevertheless, the metaboreflex contribution to VE and VE/VCO_2_ was not significantly reduced (*P* = 0.08 and *P* = 0.4).

## 4. Discussion

This study provides, for the first time, the evidence that CRT may reduce the metaboreflex contribution to the ventilatory response in a refractory heart failure population. We first demonstrate that such a device may have a peripheral effect with a significant improvement of the ventilatory response. This observation is of particular interest to understand pathophysiological basis of the response after CRT. We first suggested such a hypothesis in previous trial with a significant reduction of the peak RER and a later anaerobic threshold 6 months after CRT [8]. These observations suggested a postponed anaerobic muscular metabolism during exercise. Indeed, it is well known that patients suffering from chronic heart failure have muscular dysfunctions leading to an early anaerobic metabolism with a high carbon dioxide production, a reduction in maximal oxygen consumption and reduced effort capacities. Skeletal muscle blood flow is limited in HF population by low cardiac output and increased peripheral resistance by sympathetic tone overactivation and endothelial dysfunction [15, 16]. In addition, there is also evidence for a reduced percentage of slow-twitch type 1 fibres with oxidative enzyme content and an increased percentage of fast-twitch type 2 fibres with high glycolytic capacity [2]. Then, authors found significant reduction in oxidative enzyme activity, in mitochondrial density leading to muscular metabolites accumulation (lactates, hydrogen, prostaglandin, bradykinin etc.). These muscular changes were well correlated with an overactivation of the metaboreflex leading to high ventilatory response in heart failure [6, 7, 17].

In our investigation, we confirmed a significant improvement in oxidative capacity after CRT with an increase of the maximal oxygen consumption and a trend toward postponed anaerobic threshold. Nevertheless, the peak RER was not significantly improved. In addition, our patients experienced a significant improvement in the ventilatory response expressed as the linear regression slope of the VE/VCO_2_.

In this study, we confirmed the significant metaboreflex contribution to ventilatory parameters as VE (*P* = 0.05), RR (*P* = 0.003) and VE/VCO_2_ (*P* = 0.007) in the lower limb. Nevertheless, metaboreflex contribution to the minute ventilation was lesser than in other trials [13]. This observation is in accordance with results reported by Francis et al. [18]. Authors did not find a significant metaboreflex contribution to the VE. These results were against a significant role of muscle metaboreceptors in the genesis of the exercise hyperventilation of patients with HF. However, in this study patients probably had a less severe degree of heart failure than those in our and Scott's investigation (peak of VO_2_ of 22 ± 5 mL/min/kg versus 14 ± 4 mL/min/kg and 18 ± 2 mL/min/kg, resp.) [13]. But our findings do not contradict previous studies because of differences of protocol. In addition, metaboreflex contribution to the respiratory rate and the VE/VCO_2_ ratio during recovery appears clearly significant. These observations are in accordance with normal physiology. Unlike the minute ventilation, it is well known that RR increased during the end of exercise in normal population by anaerobic trigger. Thus, RR seems to be more regulated by muscular metabolism during exercise and then is more influenced by metareceptor trigger.

Secondly, these study provides the evidence for a significant reduction of the metaboreflex contribution to the respiratory rate and a nonsignificant reduction of the metaboreflex contribution to minute ventilation and VE/VCO_2_. These results confirmed the involvement of the peripheral muscle in the improvement of functional status after CRT. These observations are of particular interest for a better understanding of the pathophysiology of the response after implantation. Previously, Piepoli et al. found a significant reduction of the metaboreflex activation after a training protocol in a heart failure population [3]. In addition, Guazzi et al. provided evidence for a significant attenuation of the metaboreflex contribution to ventilation after cardioversion of atrial fibrillation associated with an improvement in the ventilatory response [19]. These investigations confirmed the central role of the peripheral muscular changes in the overactivation of the metaboreflex and the increase of the ventilatory response in heart failure leading to dyspnea and breathlessness during exercise and reduction of quality of life.

But how does the CRT improve the metaboreflex? It was assessed that metaboreflex activation is well correlated with the muscular perfusion [19]. Thus, CRT was confirmed to improve microcirculation [20]. In our study, we found a significant increase of the LV ejection fraction and the peak circulatory power assessing a significant improvement of hemodynamic condition after CRT. We suggest that CRT could improve peripheral blood flow by better hemodynamic conditions leading to an increase of exercise capacities. It could lead to a shift from fast-twitch type 2 fibres to slow-twitch type 1 fibres with an increase in oxidative metabolism, in mitochondrial density, in oxygen consumption and in reduction in carbon dioxide production resulting in a postponed anaerobic threshold and in a reduction of the metaboreflex activation as observed after an exercise training protocol [3, 21]. According to these results and those from Patwala et al. we could emphasize the particular interest of a systematic training program for refractory heart failure population to increase the functional status and quality of life improvement after CRT [22].

Nevertheless, this study is limited by the small population included, as well as the lack of control group. In addition, we found no significant improvement of the quality of life test, the 6-minute walking test, the duration of exercise and the maximal workload in our population. These results could be explained by the functional status of our patients: 60% of them were in class II of the NYHA status. Our patients probably had a less severe degree of heart failure than those in previous studies explaining the non significant reduction of the metaboreflex contribution to the minute ventilation. In addition, a large metaboreflex effect to arm exercise was confirmed, while a smaller effect has been observed during limb exercise. Using limb exercise and RCO, venous return may not have been completely blocked from the exercising muscle of the limb and may have underestimated the metaboreflex contribution to the ventilatory response. But according to previous studies metaboreflex evaluation using arm RCO was too painful for subjects to withstand for 3 minutes [13].

## 5. Conclusion

We first demonstrated the critical contribution of the metaboreflex reduction in the improvement of the ventilatory response (VE/VCO_2_ linear regression slope) six months after cardiac resynchronization. Whereas we found a signicant reduction of the metaboreflex contribution to the respiratory rate, we only found a trend toward reduction of the metaboreflex contribution to the VE. However, this observation is of particular interest to understand pathophysiological basis of the functional response after CRT implantation. We suggest that CRT could lead to a significant increase of exercise capacity and then to peripheral muscular remodelling with a shift from anaerobic type 2 fibres to high oxidative type 1 fibres. Nevertheless, further study including histologic data is needed to confirm this study.

## Figures and Tables

**Table 1:** Baseline characteristics. Values are given as means ± S.E.M. BNP, brain natriuretic peptid; BP, blood pressure; EF, ejection fraction; LV, left ventricle; QOL: quality of life; TDV, tele-diastolic volume; TSV, tele-systolic volume.

	Patients
*N*	10
Age	62 ± 9
Sex: male/female	9/1
Weight (kg)	79 ± 16
Systolic BP (mmHg)	114 ± 16
Heart rate (/min)	69 ± 11
NYHA status	2.4 ± 0.5
Etiology	Ischemia *n* = 4
	Idiopathic *n* = 5
	Valvular *n* = 1
QRS (ms)	147 ± 27
LV EF (%)	27 ± 4
LV TSV (mL)	142 ± 58
LV TDV (mL)	205 ± 68
6-minute walking test (meters)	460 ± 84
QOL score (Minnesota)	24 ± 14
Creatinine (micromole/L)	93 ± 13
BNP (pg/mL)	371 ± 182
Medications	
Beta blockers	9
ACE inhibitors	8

**Table 2:** Comparison of ventilatory data during rest and peak exercise with and without RCO before and 6 months after CRT. Values are given as means ± SEM. RCO, regional circulatory occlusion, RR, respiratory rate, VE, minute ventilation.

	Before CRT		After-6 month followup	
	Normal	RCO	Normal	RCO
VE at rest (L/min)	12 ± 3	13 ± 4	12 ± 3	13 ± 2
Peak VE (L/min)	51 ± 17	52 ± 14	54 ± 14	54 ± 13
Peak VCO_2_ (L/min)	1.33 ± 0.5	1.4 ± 0.4	1.5 ± 0.4	1.5 ± 0.4
RR at rest (/min)	16 ± 5	18 ± 3	18 ± 3	16 ± 3
Peak RR (/min)	30 ± 5	30 ± 5	32 ± 5	31 ± 4

**Table 3:** Comparison of exercise data before and after CRT. Values are given as means ± SEM. AT, anaerobic threshold; BP, blood pressure; CP, circulatory power. *P < 0.05 compared with baseline.

	Before CRT	After 6-month followup
Heart rate at rest (/min)	69 ± 11	70 ± 7
Peak heart rate (/min)	114 ± 25	116 ± 19
Peak systolic BP (mmHg)	150 ± 30	168 ± 31*
Maximal workload (Watts)	80 ± 25	93 ± 23
Peak VO_2_ (mL/kg/min)	14 ± 4	16.5 ± 3*
Time to AT (seconds)	163 ± 94	195 ± 69
VE at rest (L/min)	12 ± 3	13 ± 3
Peak VE (L/min)	51 ± 17	54 ± 14
Peak VCO_2_/VO_2_	1.31 ± 0.18	1.2 ± 0.1
VE/VCO_2_ slope	39 ± 10	33 ± 10*
Peak CP (mL/kg/min·mmHg)	2320 ± 821	2770 ± 791*
Duration of exercise (seconds)	474 ± 156	552 ± 142

**Table 4:** Ventilatory variables during recovery with (RCO) and without (normal) regional circulatory occlusion. Values are given as means ± SEM. ^†^ *P* = 0.05 compared with data without RCO. *P < 0.05 compared with data without RCO. ^#^ *P* < 0.05 compared with baseline.

Recovery	Parameters	Before CRT			After 6-month followup		
		Normal	RCO	Delta	Normal	RCO	Delta
1st minute	VE (l/min)	40 ± 13	43 ± 9	2 ± 7	45 ± 9	41 ± 9	−4 ± 7
	RR (/min)	27 ± 4	27 ± 4	0.7 ± 3	26 ± 3	27 ± 5	0.4 ± 3
	VE/VCO_2_	39 ± 11	39 ± 11	−0.2 ± 4	33 ± 3	34 ± 4	0.8 ± 3

2nd minute	VE (l/min)	28 ± 5	32 ± 6	4 ± 7	35 ± 6	31 ± 6	− 4 ± 6
	RR (/min)	22 ± 4	24 ± 4	2.5 ± 2	24 ± 4	23 ± 4	−0.5 ± 3
	VE/VCO_2_	40 ± 11	42 ± 11	1 ± 4	35 ± 3	37 ± 5	3.5 ± 5

3rd minute	VE (l/min)	23 ± 4	26 ± 5^†^	3 ± 4	27 ± 4	26 ± 4	−1 ± 5
	RR (/min)	19 ± 3	23 ± 3*	4.5 ± 3	22 ± 4	21 ± 3	−1 ± 3^#^
	VE/VCO_2_	41 ± 11	46 ± 11*	5.5 ± 4	36 ± 4	39 ± 6	3.5 ± 4
